# Protein corona formation on different-shaped CdSe/CdS semiconductor nanocrystals†

**DOI:** 10.1039/d4na00696h

**Published:** 2024-11-25

**Authors:** Kunisato Kuroi, Yuta Kanazawa, Akane Shinaridome, Yuna Yasuda, Minkyo Jung, Chan-Gi Pack, Fumihiko Fujii

**Affiliations:** a Faculty of Pharmaceutical Sciences, Kobe Gakuin University Kobe 650-8586 Japan kkuroi@pharm.kobegakuin.ac.jp fumihiko.fujii@pharm.kobegakuin.ac.jp; b Neural Circuit Research Group, Korea Brain Research Institute Daegu 41062 Korea; c Convergence Medicine Research Center (CREDIT), Asan Institute for Life Sciences, Asan Medical Center Seoul 05505 Korea; d Department of Biomedical Engineering, University of Ulsan College of Medicine Seoul 05505 Korea

## Abstract

Nanoparticles (NPs) have been widely studied and applied in medical and pharmaceutical fields. When NPs enter the *in vivo* environment, they are covered with protein molecules to form the so-called “protein corona”. Because NPs and proteins are comparable in size, the shape of NPs has a significant impact on NP–protein interactions. Although NPs of various shapes have been synthesized, how the shape of NPs affects the protein corona is poorly understood, and little is known about the underlying molecular mechanism. In the present study, we synthesized spherical, football-shaped, and rod-shaped semiconductor nanocrystals (SNCs) as model NPs and compared their interaction with human serum albumin (HSA) using fluorescence correlation spectroscopy, fluorescence quenching, Fourier-transform infrared spectroscopy, and thermodynamic analysis. Based on the binding enthalpy and entropy and secondary structural changes of HSA, with the help of hydrodynamic diameter changes of SNCs, we concluded that HSA adopts a conformation or orientation that is appropriate for the local curvature of SNCs. This study demonstrates the effect of NP shape on their interaction with proteins and provides a mechanistic perspective.

## Abstract 2

Nanoparticles in biological environments adsorb proteins, forming a “protein corona”. This study investigates how nanoparticle shape influences the formation of the protein corona using differently shaped semiconductor nanocrystals.

## Graphical abstract

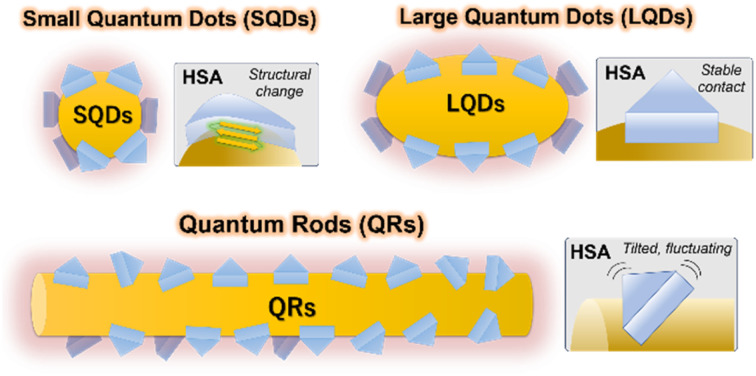

## 1. Introduction

Nanoparticles (NPs) are generally less than 100 nm in size and have great application potential in medicine and pharmaceuticals.^1^ Once NPs enter the human body, they are always interacting with protein molecules in biofluids and become covered with proteins. This protein layer formed on the NP surface, termed “protein corona”, has attracted much interest because it affects the physicochemical properties of NPs or their designed functionalities.^2,3^ Detailed molecular-level insights into the interaction between NPs and proteins are important for understanding and controlling the protein corona. Hence, this subject has been studied for more than a decade.^4–6^

Because NPs and proteins have similar sizes, the size and morphology of NPs should significantly affect NP–protein interactions and hence protein corona formation. Indeed, a series of studies using spherical NPs of various sizes have established that the NP–protein interactions tend to be stronger for larger NPs.^7–10^ Meanwhile, NPs of various shapes have also been synthesized with expected applications in life science due to their unique physical or optical properties.^11^ For example, quantum nanorods could track the rotational motion of single protein molecules,^12^ and gold nanorods could be used in cancer treatment.^13^ The effects of morphology of NPs on their interaction with biological molecules cannot be ignored for *in vivo* applications. Indeed, several studies have demonstrated the impact of NP shape on the protein corona. The amount of proteins and type of protein adsorbed on NPs after incubation in serum or *in vivo* differed significantly depending on the NP shape.^14,15^ It has also been shown that the NP shape affects the physicochemical properties of adsorbed proteins, such as the binding strength, secondary structure, and enzymatic function.^16–21^

However, there is scarce mechanistic understanding of the morphological effects of NPs on their interactions with proteins. Clarifying the structure and orientation of bound proteins is necessary for this purpose. Fluorescence correlation spectroscopy (FCS) is a possible way for discussing the orientation of bound proteins. Because FCS can sensitively detect subtle changes in the hydrodynamic diameter of solute molecules,^22^ it has been applied to study interactions between biological molecules or NPs, as exemplified by our previous research studies.^23–25^ By tracking the increase in the NP diameter upon protein corona formation, Nienhaus *et al.* showed that proteins were adsorbed as a monolayer in most cases and that their orientation depended on the surface charge state of both the proteins and NPs.^26–28^ A similar approach using scattering correlation spectroscopy was recently applied to differently shaped gold NPs, and the shape-dependent orientations of bound proteins were discussed.^29^

Thermodynamic analysis is another useful way to comprehensively understand the binding mechanism of proteins to materials, including the roles of chemical bonds and solvent water molecules.^30^ For example, for NP–protein interactions, a negative binding enthalpy has been attributed to the formation of noncovalent bonds, whereas a positive binding entropy has been attributed to the release of solvating water molecules from the contact surface.^31,32^ Calorimetric techniques such as isothermal titration calorimetry (ITC)^9,19,31,33^ and temperature-dependent measurements^8,17,32,34^ have also been widely applied to study the protein corona from a thermodynamic perspective. Recently, based on the binding enthalpy and entropy, a two-step binding mechanism (*i.e.*, association concomitant with desolvation, followed by the formation of chemical bonds) was proposed for NP–protein interactions.^32,35^

In this study, a mechanistic view of the effect of NP shape on NP–protein interaction was obtained by combining FCS and thermodynamic analysis, with the help of infrared (IR) spectroscopy which we have previously used to elucidate the surface state of NPs^36^ and the structure and dynamics of proteins.^37,38^ For the NPs, we synthesized three types of semiconductor nanocrystals (SNCs) of different shapes: small quasi-spherical ones (small quantum dots, SQDs), large football-shaped ones (large quantum dots, LQDs), and rod-shaped ones (quantum rods, QRs). SNCs have excellent optical properties such as a large absorption cross section, high quantum yield, and high resistance to photobleaching,^39^ all of which facilitate the application of the FCS technique. Furthermore, owing to their large Stokes shifts, fluorescence resonance energy transfer (FRET) has also been used to investigate protein corona formation.^40^ Thus, SNCs are appropriate model NPs for studying protein coronas using spectroscopic techniques. For the protein, we selected human serum albumin (HSA) because it is the most abundant protein in blood and has often been used to study protein coronas. Based on the thermodynamic parameters (binding enthalpy and entropy), thickness of the protein corona estimated from FCS, and secondary structural changes of HSA from IR spectroscopy, SNC-shape dependent binding modes of HSA were finally proposed.

## 2. Methods

### 2.1. Materials

The following reagents were purchased and used as received for the synthesis of SNCs. Selenium (Se, >99.99%), cadmium oxide (CdO, >99.99%), and oleic acid (OA, 90%) were purchased from Sigma-Aldrich. Tri-*n*-octylphosphine oxide (TOPO), tri-*n*-octylphosphine (TOP), hexylphosphonic acid (HPA, >98%), and octadecylphosphonic acid (ODPA, >98%) were purchased from Tokyo Kasei, Japan. Sulfur (S, >99.99%) was purchased from Stream Chemicals. Reduced glutathione (GSH) and potassium *t*-butoxide (*t*-BuOK) were purchased from Fujifilm-Wako, Japan, and used as received to solubilize the SNCs.

HSA (F-V) was purchased as a crystalline powder from Nacalai Tesque, Japan, and dissolved at the desired concentration in phosphate buffered saline (PBS; NaCl 137 mM, KCl 2.7 mM, Na_2_HPO_4_ 10 mM, KH_2_PO_4_ 1.76 mM, pH 7.4) or water. The concentration of HSA was determined spectroscopically using its absorption coefficient (*ε*_278_ = 37 000 cm^−1^ M^−1^).^40^ The prepared HSA solution was divided into *ca.* 500 μL aliquots for a single experiment, snap-frozen using liquid nitrogen and stored at −80 °C until use.

### 2.2. Preparation of SNCs

SQDs, QRs, and LQDs with different morphologies were synthesized as CdSe/CdS core–shell type SNCs by controlling the growth of the CdS shell on the CdSe core. Their synthesis procedures were based on that by Deka *et al.*^41^ (for QRs) or Cirillo *et al.*^42^ (for SQDs and LQDs) with modifications. The SNCs were synthesized using the hot-injection method in a 3-neck flask under an Ar atmosphere with constant stirring.

First, to synthesize the CdSe cores, a mixture of CdO (0.06 g), TOPO (3.00 g), and ODPA (0.28 g) in a 3-neck flask was incubated at 150 °C for 45 min under Ar gas flow to thoroughly replace the air and then heated until the solution became colorless (*ca.* 300 °C). Next, TOP (1.8 mL) was injected, and the mixture was further heated to 370 °C. Se (0.058 g) dissolved in TOP (0.434 mL) was rapidly injected into the mixture, followed by incubation for 30 s and rapid cooling with ice water. The CdSe product was precipitated by adding excess methanol, redispersed in 4 mL of TOP, and stored in the dark. The concentration of CdSe particles was determined to be *ca.* 150 μM using the reported absorption coefficient of *ε*_350_ = 0.6 × 10^6^ cm^−1^ M^−1^.^43^

To synthesize the SQDs, a mixture of CdO (0.09 g), TOPO (3.00 g), and OA (4 mmol, *ca.* 1.26 mL) in a 3-neck flask was incubated at 120 °C for 1 h under Ar gas flow and then further heated until the solution became colorless (*ca.* 300 °C). After adding TOP (1.8 mL), the temperature was increased to 330 °C, and the mixture was rapidly injected with a solution containing the CdSe core particles (87 nmol, 592 μL of the stock solution) and S (0.090 g) in TOP (1.4 mL). The mixture was incubated for 20 s and rapidly cooled with ice water. After annealing at 100 °C for 1 h, the reaction was quenched by adding 10 mL anhydrous toluene. The SQD product was precipitated by adding excess methanol. The precipitate was re-dissolved in toluene and reprecipitated with methanol. This purification step was repeated twice. The final product was dissolved in 2 mL cyclohexane and stored in the dark. The LQDs were synthesized in exactly the same manner as that of SQDs, except that 35.7 nmol of CdSe core particles were used and the incubation time at 330 °C was extended to 3 min for further growth of the CdS shell.

The QRs were similarly synthesized using HPA and ODPA for growing the CdS shell along the long axis. A mixture of CdO (0.06 g), TOPO (3.00 g), ODPA (0.280 g), and HPA (0.080 g) in a 3-neck flask was incubated at 100 °C for 1 h under Ar gas flow and heated until the solution became colorless (*ca.* 300 °C), followed by the addition of 1.8 mL TOP. After the temperature was increased to 350 °C, a solution containing the CdSe core particles (24.7 nmol, 168 μL of the stock solution) and S (0.120 g) in 1.8 mL TOP was rapidly injected into the mixture and incubated for 120 s. Then, the reaction mixture was rapidly cooled with ice water, annealed at 100 °C for 1 h, and quenched by adding anhydrous toluene (1 mL). The procedures for purifying and storing the product were the same as that used for the SQDs or LQDs.

The synthesized SQDs, LQDs, and QRs were solubilized in water by capping their surfaces with GSH, according to the procedure by Jin *et al.*^44^ with modifications. A 33 μL aliquot was withdrawn from the SNC stock solution in cyclohexane, dried in air flow, and re-dissolved in tetrahydrofuran (THF, 500 μL). After centrifugation (16 000×*g*, 5 min), a 400 μL aliquot was withdrawn from the supernatant and mixed with aqueous GSH solution (390 mM, 200 μL). The mixture was incubated for 5 min at 70 °C, vortexed for 30 s, and then sonicated for 30 s. This cycle of vortexing and incubation was repeated three times. The resultant precipitate was washed with THF/water (2 : 1 v/v, 600 μL) and dissolved in 400 μL water, followed by the addition of *t*-BuOK (3.3 mg). After centrifugation (16 000×*g*, 5 min) to remove the aggregates, the supernatant was collected as a water-solubilized SNC solution.

The concentration of solubilized SNCs was determined from the UV-Vis absorption spectrum, assuming that the CdSe peak at around 600 nm (see Fig. 2(a)) has an absorption coefficient of *ε* = 3.0 × 10^5^ cm^−1^ M^−1^ regardless of the NP shape. This value was reported by Gong *et al.*^45^ for CdSe/CdS quantum dots with similar core absorption (∼590 nm). Such an assumption is valid because the spectral profile of the core absorption band was not significantly affected by the shape of the SNCs. Indeed, the spectra of SQDs and QRs in this study almost overlapped in the CdSe absorption region.

### 2.3. Transmission electron microscopy (TEM)

Solubilized SNC samples were applied to a PD-10 desalting column (Cytiva) to replace the solvent with pure water. About 5 μL of each sample was dropped on a carbon-coated copper TEM grid (FF200-Cu, Electron Microscopy Science, USA), and excess solution was removed with filter paper. Then, the sample was allowed to dry prior to analysis. TEM images were obtained using an electron microscope at an acceleration voltage of 120 kV and 2048 × 2048 pixels at 80 000× magnification (Tecnai G2, Thermo Fisher Scientific, Waltham, MA, USA) and recorded using Gatan Microscopy Suite® 2.0 software (Gatan, Japan). For each type of SNC, the long- and short-axis lengths were manually measured using ImageJ software for more than 150 particles in the image. The measured lengths were then subjected to statistical analysis.

### 2.4. Dynamic light scattering (DLS) measurements

DLS measurements were performed using a Zetasizer Nano ZSP (Malvern) equipped with a 632 nm He–Ne laser. Scattered light was collected at a 173° angle. The solubilized SNC sample was first diluted using PBS by about 100 times. Prior to measurements, the sample solution was sonicated and passed through a 0.25 μm pore membrane filter before placement in a polystyrene cuvette. As measurement parameters, the refractive index (*n*) and extinction coefficient (*k*) of the solute SNCs at 632 nm were assumed to be those of CdSe (*n* = 2.6 and *k* = 0.30).^46^

Zeta potentials (*ζ*) of solubilized SNCs or HSA were measured using the same DLS equipment and a capillary zeta cell (DTS1070, Malvern). Prior to measurements, sample solvent was exchanged with ultrapure water (Milli-Q) to avoid interference from ionic strength. Sample solutions were similarly filtered as described above.

### 2.5. Spectroscopic measurements

Fluorescence spectra were recorded on an RF-6000 spectrometer (Shimadzu) in a quartz cuvette (3 mm path length). The excitation wavelength was set at 480 nm (for SNCs) or 295 nm (for HSA). UV-Vis absorption spectra were recorded using a V-660 spectrometer (Jasco) in the same cuvette. Spectral band widths were set to 2 nm in absorption measurements and 5 nm for both excitation and emission in fluorescence measurements.

For fluorescence quenching experiments, the solubilized SNC solution was titrated in steps of 2 μL into 300 μL of HSA solution in PBS (2.8 μM), mixed gently, and incubated for several minutes. In the quenching experiment, the inner filter effect was considered, and sample absorbance at the excitation wavelength (295 nm) was set to no more than 0.16 in each case, which ensured that the inner filter effect was almost negligible. The temperature of the cuvette was controlled in the range of 283–323 K using a thermojacket with circulating water from a thermostat bath (EYELA), and the temperature was confirmed using a thermocouple immediately before measurements. Each experiment was repeated at least three times to ensure reproducibility.

The IR spectra were recorded on an FTIR-8400S spectrometer (Shimadzu) equipped with a deuterated triglycine sulfate (DTGS) detector under dry air flow. IR spectra were recorded at a resolution of 4 cm^−1^ and typically averaged 64 times. Atmospheric signals (vapor or CO_2_) were corrected using Shimadzu IRsolution software. For the Fourier-transformed infrared (FTIR) samples, ultrapure water (Milli-Q) was used as the solvent for both HSA and SNCs. In the case of solubilized SNCs, because the final solvent of SNCs contains *t*-BuOK in their solubilization process, the solvent was exchanged with pure water by several cycles of centrifugation, followed by dilution with water using a 10 kDa (MWCO) spin filter. A 5 μL aliquot of sample solution was placed on a 25 mm diameter CaF_2_ plate (2 mm thickness) and dried. The sample film formed on the CaF_2_ plate was sandwiched between two plates with a 2 mm thick silicon rubber spacer mounted in a transmission cell holder. For measurement of the HSA–SNC complex, the molar ratio of HSA to SNCs was set to 10 : 1 (1000 pmol of HSA and 100 pmol of SNCs), and the mixture was dried.

Circular dichroism (CD) spectra were recorded on a J-725 CD spectrometer (Jasco) under N_2_ gas flow. The solvent of SQDs and HSA solutions was replaced with PBS using a PD-10 column. For measurements of the HSA–SQD complex, the final concentrations of HSA and SQDs were adjusted to 2 μM and 40 μM, respectively. CD spectra were measured using a 10 μm pathlength quartz cell, assembled from two quartz plates. A 20 μL aliquot of the mixed solution was placed on one plate, and the other plate with a 10 μm depth groove was placed on top to encapsulate the solution. A 40 μM HSA solution in PBS was similarly measured. The CD spectrum of PBS was also measured in the same manner as that of a control to subtract the subtle contribution of the quartz cell or solvent. CD spectra were subjected to the secondary structure analysis by using the open access software BeStSel (https://bestsel.elte.hu/index.php).^47^

### 2.6. Fluorescence correlation spectroscopy (FCS)

FCS measurements were performed using a confocal laser scanning microscope (Fluoview FV1000, Olympus) with a 60× objective (NA = 1.2). A 20 μL sample was dropped at the center of each well in an 8-well chambered cover glass placed on the microscope. A 405 nm diode laser (*ca.* 5 μW) was used to excite the SNCs and HSA samples. Fluorescence was collected using the same objective lens in the single-photon counting mode and subsequently analyzed using a correlator equipped in the microscope. The pinhole size of the microscope was automatically set typically at 120 μm in FCS measurements. The measurements were repeated at least three times at the same focal point, and three autocorrelation curves were obtained and averaged to improve the signal-to-noise ratio. Prior to the measurement of SNCs and HSA, the parameters of the apparatus were determined using a reference dye (Rhodamine 6G) with a 473 nm diode laser, as described in the later Section 3.3. The SNC solution was mixed with HSA at various concentrations in PBS and incubated for several minutes before measurements. The concentration of SNCs was set to 17 nM (for SQDs and LQDs) or 8.3 nM (QRs). The experiments including sample preparation were repeated at least three times to ensure reproducibility.

## 3. Results and discussion

### 3.1. Size and shape of the SNCs

**Fig. 1:**
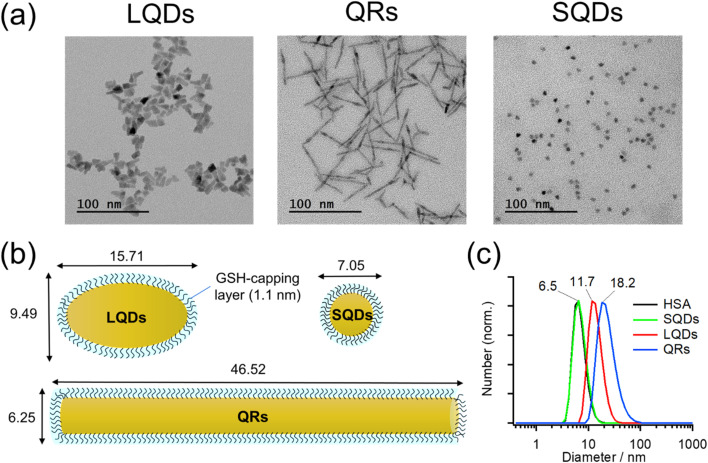
(a) TEM images of the LQDs, QRs, and SQDs. (b) Schematic of GSH-capped SNCs. The GSH capping layer is assumed to have a thickness of 1.1 nm. (c) Normalized size distributions of SNCs as determined by DLS. The result of HSA is also included for comparison.

Typical TEM images of the LQDs, QRs, and SQDs are shown in Fig. 1(a).

Morphologies of LQDs, QRs, and SQDs were football-like, rod-like, and quasi-spherical, respectively. Statistical analysis of approximately 150 SNC particles revealed the distribution patterns of the long axis (length, *L*), short axis (width, *W*), and aspect ratio (*p* = *L*/*W*) for the LQDs and QRs (Fig. S1(a)–(c) and (d)–(f) in the ESI,† respectively). The distribution pattern of the diameter (*d*) of the SQDs is shown in Fig. S1(g), ESI.† The histograms show that the SNC particles were uniform in size. The average dimensions of the SNCs were determined from the TEM images and are shown in Table S1, ESI.†

In this study, the SNCs were capped with GSH for solubilization in water. The thickness of this capping layer was estimated geometrically from the length of the nine C–C bonds in GSH (bond angles of 109.5°) extending from the sulfur atom attached to the SNC surface: 9 × 0.15 × sin(109.5°/2) = 1.1 nm. Then, the size and shape of the solubilized SNCs were calculated by simply adding twice of this thickness to the dimensions of bare SNCs (Table S1†), as shown in Table 1 and schematically depicted in Fig. 1(b).

**Table 1:** Dimensions of GSH-capped LQDs, QRs, and SQDs

	QRs	LQDs	SQDs
*W*/nm	6.25 ± 0.52	9.49 ± 1.51	7.05 ± 0.60
*L*/nm	46.52 ± 6.60	15.71 ± 2.60	—
*p* = *L*/*W*	7.48 ± 1.11	1.69 ± 0.39	—
Surface area/nm^2^	977 ± 169	416 ± 99	157 ± 27

The surface areas of the SNCs in Table 1 were calculated mathematically by assuming the LQDs as prolate ellipsoids, the QRs as cylinders, and the SQDs as spheres.

Fig. 1(c) shows the size distributions of the SNCs in solution as measured by DLS. From the results, the SNCs were monodispersed in solution and the samples were of good quality. The hydrodynamic diameters (*d*_H_) of SQDs, LQDs, and QRs were 6.5, 11.7, and 18.2 nm, respectively. The result of HSA was also included for comparison, demonstrating that the SQDs and HSA have almost the same size. The *d*_H_ of SQDs (6.5 nm) is close to their diameters (*W* = 7.05 nm, Table 1) determined from TEM images. For the non-spherical LQDs and QRs, by substituting *L* and *p* in Table 1 into the modified Stokes–Einstein equation (eqn (S3) in Section S2, ESI†), their hydrodynamic diameters were calculated to be 11.48 nm for the LQDs and 17.10 nm for the QRs. These values are also in good agreement with the results from DLS measurements (11.7 nm for the LQDs and 18.2 nm for the QRs). Thus, the dimensions of the SNCs in Table 1 represent their particle shapes in solution.

Zeta potentials (*ζ*) of GSH-capped SNCs were determined in DLS measurements: *ζ*_SQDs_ = −31.9 ± 3.6 mV, *ζ*_QRs_ = −28.9 ± 2.0 mV, and *ζ*_LQDs_ = −33.4 ± 2.3 mV. The negative zeta potentials indicate that the GSH ligands on each SNC are deprotonated, which was confirmed by IR spectroscopy in the following section. The surface charge density of the SNCs was also found to be similar based on the magnitude of their zeta potentials. Zeta potential of HSA was also confirmed to be negative (*ζ*_HSA_ = −14.5 ± 3.0 mV), which will contribute to the formation of a negatively charged HSA–SNC complex with the expected colloidal stability.

### 3.2. Spectroscopic properties of SNCs

**Fig. 2:**
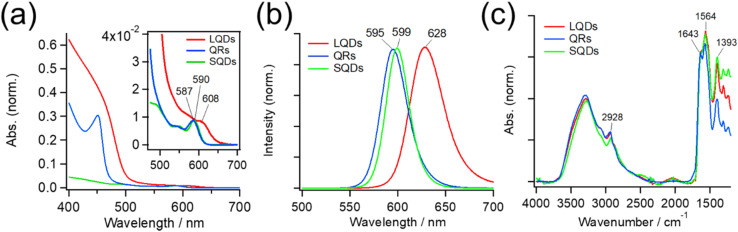
(a) UV-Vis absorption spectra of the LQDs, QRs, and SQDs. The spectra are normalized by the absorption peak of the CdSe core (inset) around 600 nm. (b) Peak-normalized fluorescence spectra of the LQDs, QRs, and SQDs. (c) IR absorption spectra of the LQDs, QRs, and SQDs. The spectra are normalized by the amide I band (∼1643 cm^−1^) of surface GSH ligands.

Fig. 2(a) shows absorption spectra of the SNCs. The weak absorption band at approximately 600 nm in each spectrum originates from the CdSe core, and it generally shifts to a longer wavelength when the core diameter increases or its surrounding shell becomes thicker.^45,48–50^ Because SNCs in the present study were synthesized from the same CdSe core, the red-shifted core absorption of the LQDs (608 nm) suggests thicker shells compared to those of the SQDs and QRs. The stronger absorption at shorter wavelengths (<*ca.* 500 nm) originated from the CdS shell, and its spectral profile depended on the shell structure. The QRs and LQDs show a much higher relative intensity of CdS absorption than the SQDs, indicating that the former two have larger CdS shells. In particular, the sharp absorption peak at around 450 nm in QRs is characteristic of CdSe/CdS nanorods.^48,49^ The rod width of bare QRs can be calculated by drawing a tangent around the inflection point of this peak (see Section S3, ESI† for details).^48^ The calculated width of 4.33 nm is also close to that obtained from the TEM images (*W* = 4.05 nm, Table S1†). These spectral observations support the particle shapes determined by TEM.

Fig. 2(b) shows the peak-normalized fluorescence spectra of the SNCs. Consistent with the red-shifted core absorption of the LQDs, the fluorescence spectrum of the LQDs was also red-shifted by approximately 30 nm compared with the other SNCs. From the fluorescence intensities and UV-Vis absorption of the SNCs, the quantum yield (QY) was determined to be 0.47 for the SQDs, 0.35 for the QRs, and 0.06 for the LQDs (see Section S4, ESI† for details).

Fig. 2 (c) also shows the IR spectra of SNCs normalized by the peak intensity at ∼1643 cm^−1^. Most of the spectral bands originate from GSH ligands on the surface, which show the amide I band (∼1643 cm^−1^) and asymmetric stretching band of carboxylate (1564 cm^−1^).^36^ The peak at 1393 cm^−1^ may be assigned to the methylene stretching mode of GSH. The minor peak at 2928 cm^−1^ was due to the C–H stretching mode of residual TOPO ligands that were not replaced by GSH. The similar IR spectra of SQDs, QRs, and LQDs suggest that their surface ligands are in almost the same state. Hence, any surface differences among these SNCs can be ignored when considering their interaction with HSA.

### 3.3. Detection of protein corona formation by FCS

**Fig. 3:**
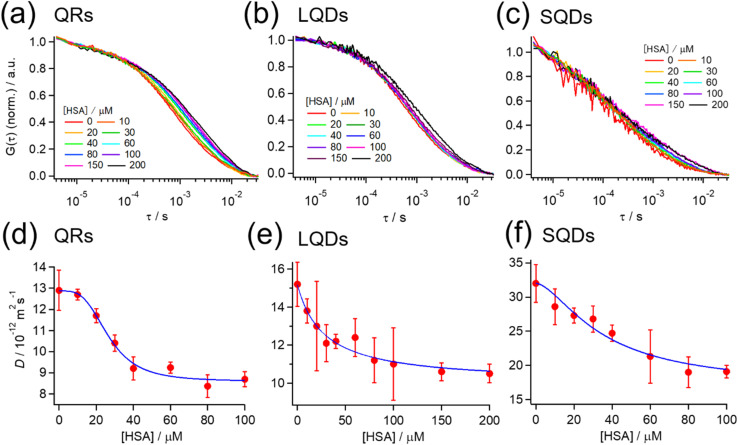
(a–c) Normalized autocorrelation function (*G*(*τ*)) at various HSA concentrations from FCS measurements for (a) QRs, (b) LQDs, and (c) SQDs. *G*(*τ*) is normalized by the average intensity at *τ* < 10^−5^ s. (d–f) Effect of the HSA concentration on the diffusion coefficient (*D*) of (d) QRs, (e) LQDs, and (f) SQDs. Blue solid line: the fitting curve obtained using eqn (2).

Fig. 3(a)–(c) show the normalized autocorrelation function (*G*(*τ*)) for each type of SNC, obtained from the FCS measurements at various HSA concentrations. For all SNCs, the correlation curve decayed to the baseline at *τ* > ∼10^−4^ s because of the diffusion of SNCs. For each type of SNC, the decay rate clearly became slower with the increasing HSA concentration, indicating the slowing down of SNC diffusion due to the binding of HSA. These results confirm the formation of a protein corona by HSA on each type of SNC. A minor fast decay of *G*(*τ*) (*τ* < ∼10^−4^ s) independent of the HSA concentration was also observed for each type of SNC. This decay component was assigned to the photodynamics of SNCs, as reported previously.^51^

The *G*(*τ*) curves of SNCs at various HSA concentrations were fitted using eqn (1) below, which assumes a three-dimensional Gaussian intensity profile of the excitation laser in the confocal volume with a lateral radius *W*_0_ and an axial radius *W*_*z*_.^22,51^1
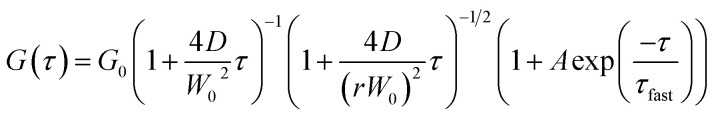
In eqn (1), the first two terms enclosed by parentheses represent the diffusion of SNCs. *G*_0_ is the initial amplitude, *D* is the diffusion coefficient of SNCs, and *r* = *W*_*z*_/*W*_0_. We chose the fixed values of *W*_0_ = 2.5 × 10^−7^ m and *r* = 6 from measurements performed on a reference dye (Rhodamine 6G) with a known diffusion coefficient (*D* = 2.8 × 10^−10^ m^2^ s^−1^).^52^ The last term in eqn (1) enclosed by parentheses corresponds to the fast decay phase of *G*(*τ*) due to the photo-dynamics of SNCs, and this term is considered trivial in this study. The autocorrelation curves in Fig. 3(a)–(c) are well reproduced using eqn (1), as shown by the typical fitting results in Fig. S4, ESI.† The *D* value of each type of SNC was determined by the fitting and plotted against the HSA concentration, as shown in Fig. 3(d)–(f). The results show that *D* decreased with the HSA concentration as expected, which in turn indicates an increase in the hydrodynamic diameter of SNCs according to the Stokes–Einstein relationship.

Assuming that the binding of HSA to SNCs is described by the Hill equation with an apparent dissociation constant *K*_D_ and Hill coefficient *n*, the *D* value of SNCs at [HSA] = *x* μM (*i.e.*, *D*(*x*)) can be described as follows (for the derivation of eqn (2) see Section S6, ESI†):2
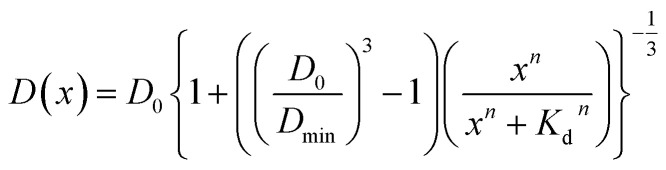
where *D*_0_ and *D*_min_ are the initial and minimum (final) diffusion coefficients of the SNCs, respectively. The experimental data in Fig. 3(d)–(f) are well fitted using eqn (2), as shown by the continuous blue lines. The fitting parameters (*K*_D_, *n*, *D*_0_, and *D*_min_) for each type of SNC were determined and are summarized in Table 2 (rows 1–4). *K*_D_ was in the range of 10^−5^ M and consistent with previous FCS studies of the protein corona.^34,51^ The different *K*_D_ values in Table 2 may suggest a particle shape effect. However, it is difficult to discuss their differences because of the large standard deviation of *K*_D_ for the LQDs and SQDs, which may be attributed to the lower signal-to-noise ratio of their correlation curves (Fig. 3(a)–(c)), resulting from the much smaller quantum yield of LQDs or much smaller absorption cross-section of SQDs compared to QRs. The particle shape effect may also be reflected in different Hill coefficient (*n*) values, which indicate positive (*n* > 1) or negative (*n* < 1) binding cooperativity. The SQDs and QRs showed positive cooperativity, whereas the LQDs showed non-cooperativity. The different binding cooperativities of the three types of SNCs imply a shape-dependent binding mechanism for HSA.

**Table 2:** Parameters obtained from the FCS experiments. Rows 1–4: binding parameters for each type of SNC according to eqn (2). Rows 5–7: initial and maximum hydrodynamic diameters of the SNCs (*d*_0_ and *d*_max_) and the thickness (Δ*d*) of the HSA layer

	QRs	LQDs	SQDs
*K*_D_/μM	34 ± 4	63 ± 56	76 ± 76
Hill coefficient (*n*)	3.5 ± 0.9	1.0 ± 0.4	1.7 ± 0.7
*D*_0_/10^−11^ m^2^ s^−1^	1.3	1.5	3.2
*D*_min_/10^−11^ m^2^ s^−1^	0.86	1.0	1.7
*d*_0_/nm	18.2	11.7	6.5
*d*_max_/nm	27.4	17.8	12.2
Thickness (Δ*d*)/nm	4.6	3.2	2.9

Furthermore, the thickness of the HSA adsorption layer (Δ*d*) was estimated as follows. Suppose that the initial and maximum (final) hydrodynamic diameters are *d*_0_ and *d*_max_, respectively. *d*_max_ can be calculated as *d*_max_ = *d*_0_ (*D*_0_/*D*_max_). Using the hydrodynamic diameters of each type of SNC obtained from DLS measurements (Fig. 1(c)) for *d*_0_, *d*_max_ was calculated and is shown in Table 2. Then, Δ*d* for the SQDs was simply calculated as (*d*_max_ − *d*_0_)/2 = 2.9 nm. In the case of LQDs or QRs, Δ*d* was determined by solving the modified Stokes–Einstein equation (eqn (S3)), where *d*_H_ was replaced by *d*_max_ and the long- and short-axis lengths (*L* and *W*) were replaced with those with additional layer thickness (*i.e.*, *L* + 2Δ*d* and *W* + 2Δ*d*). In this way, Δ*d* was determined to be 4.6 nm for the QRs and 3.2 nm for the LQDs. The obtained Δ*d* values are summarized in Table 2. The structure of HSA can be approximated using an equilateral triangular prism with a size of 8 nm and a height of 3 nm.^51,53,54^ Therefore, the thickness of the protein corona layer (Δ*d*) on each type of SNC was comparable with the height of HSA. Hence, we expect the monolayer adsorption of HSA. Many other studies of protein corona also predicted such monolayer adsorption of proteins onto NPs.^55^ These thickness values of SNCs will be discussed later in detail from the viewpoint of HSA orientation.

The number of HSA molecules can also be estimated from this thickness (Δ*d*) as follows. Using dimensions of SNCs in Table 1 (*L* or *W*), the initial particle volume (*V*) was calculated geometrically; *V*_QRs_ = 1427 nm^3^, *V*_SQDs_ = 183 nm^3^, and *V*_LQDs_ = 741 nm^3^. After HSA adsorption, the increased particle volume (*V*′) is calculated considering the increased dimensions by the thickness (*i.e.*, *L* + 2Δ*d* and *W* + 2Δ*d*) as follows: 
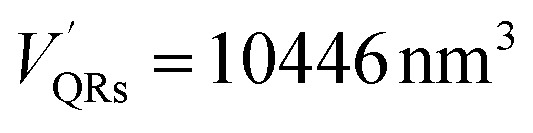
, 
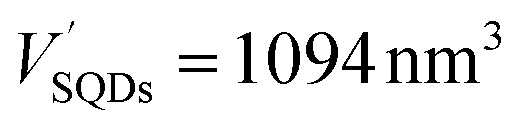
, and 
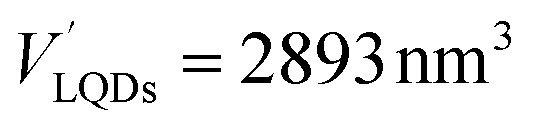
. By dividing this volume difference (Δ*V* = *V*′ − *V*) by the geometrical volume of HSA (*V*_HSA_ = 97 nm^3^, assuming the equilateral triangular prism shape described before), the number of adsorbed HSA molecules (*N*) for each SNC was estimated using *N* = Δ*V*/*V*_HSA_. The calculated values were *N*_QRs_ = 93, *N*_SQDs_ = 9.4, and *N*_LQDs_ = 22.

### 3.4. Fluorescence quenching of HSA by SNCs and the thermodynamic approach

So far, we confirmed the formation of protein corona by HSA on SNCs using FCS measurements. Next, we further examine and compare the binding of HSA to differently shaped SNCs by utilizing intrinsic Trp fluorescence of HSA. HSA has one Trp residue (Trp215), and fluorescence quenching by the addition of NPs has been widely utilized to investigate NP–protein interactions.^8,17,32,56,57^

**Fig. 4:**
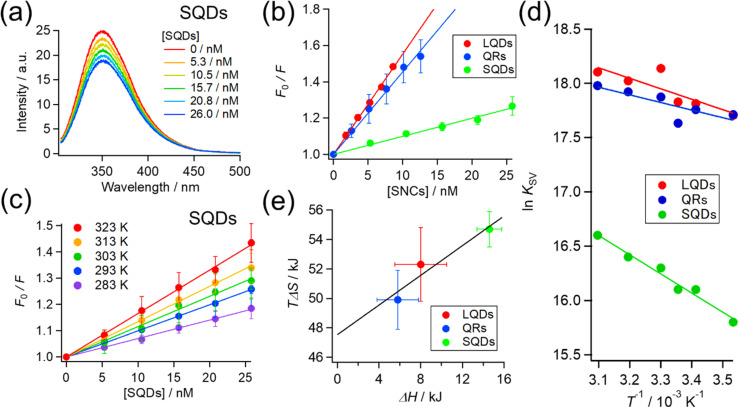
(a) Fluorescence spectra of HSA quenched by SQDs at room temperature (298 K). Each spectrum was the average over at least 3 independent experiments. (b) Stern–Volmer plot for each type of SNC (at 298 K). (c) Stern–Volmer plots for SQDs at various temperatures. (d) van't Hoff plot for each type of SNC from the temperature dependence of the Stern–Volmer constant (*K*_SV_). (e) Plots of enthalpy (Δ*H*) *vs.* entropy (*T*Δ*S*) for the binding of HSA with each type of SNC.

The HSA fluorescence was gradually quenched by titrating with the SQDs, QRs, or LQDs. Fig. 4(a) shows the changes in HSA fluorescence for the SQDs. The corresponding results for the QRs and LQDs are respectively shown in Fig. S5(a) and (b), ESI.†

The fluorescence quenching results were analyzed using the Stern–Volmer equation:3
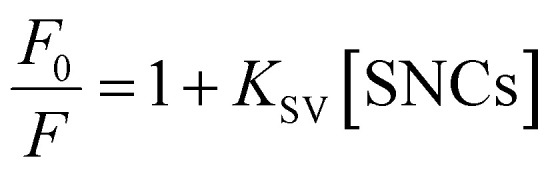
where *F*_0_ and *F* are the HSA fluorescence intensities without and with SNCs, respectively, and *K*_SV_ is the Stern–Volmer constant. Notably, *K*_SV_ can be regarded as the binding constant between the SNCs and HSA, as will be explained later. The Stern–Volmer plot (*F*_0_/*F vs.* [SNCs]) is shown in Fig. 4(b), where the slope of the linear fitting line using eqn (3) equals *K*_SV_. The *K*_SV_ values determined from the plots were 0.99 × 10^7^ M^−1^ (SQDs), 4.5 × 10^7^ M^−1^ (QRs), and 5.5 × 10^7^ M^−1^ (LQDs), as summarized in Table 3. Considering the small standard deviations in the Stern–Volmer plot of the LQDs (<0.05, almost invisible in the plot), the difference in *K*_SV_ between the LQDs and QRs was regarded as valid.

**Table 3:** Stern–Volmer constants (*K*_SV_) and thermodynamic parameters (*T* = 298 K) for the binding of HSA onto SNCs determined from fluorescence quenching experiments

	QRs	LQDs	SQDs
*K*_SV_/10^7^ M^−1^	4.5	5.5	0.99
Δ*G*/kJ mol^−1^	−44	−44	−40
Δ*H*/kJ mol^−1^	5.8 ± 2.0	8.0 ± 2.5	15 ± 1.2
*T*Δ*S*/kJ mol^−1^	50 ± 2.0	52 ± 2.5	55 ± 1.2

The *K*_SV_ values in Table 2 are in the order of 10^7^, ensuring that the quenching process is the “static” type. Therefore, *K*_SV_ can be regarded as the binding constant between HSA and SNCs. To be specific, there are “dynamic” and “static” types of fluorescence quenching processes.^58^ Fluorescence quenching of the former type is caused by collisions between fluorophores in the excited state and quencher molecules. In the latter type, this is caused by the binding of quenchers to fluorophores already in the static ground state. *K*_SV_ in dynamic quenching can be expressed using *K*_SV_ = *k*_q_*τ*, where *τ* is the fluorescence lifetime (typically about 10^−8^ s for Trp fluorescence) and *k*_q_ is the quenching rate constant that should not exceed the diffusion-limited value (typically 10^8^–10^9^ M^−1^ s^−1^ in solution).^58^ Therefore, if the quenching process is “dynamic”, *K*_SV_ should be approximately less than 10^−8^ × 10^9^ = 10^1^ M^−1^, which is distinctly lower than our values. Hence, we consider the fluorescence quenching of HSA observed here to be the “static” type and the *K*_SV_ value to be the binding constant.

The *K*_SV_ of the SNCs with HSA followed the order LQDs > QRs > SQDs. Presumably, a particle with a larger surface area is expected to have a higher binding constant owing to its increased loading capacity. However, the order of *K*_SV_ does not match that of their surface areas, which is QRs (977 nm^2^) > LQDs (416 nm^2^) > SQDs (157 nm^2^), as shown in Table 1. Therefore, the differences in *K*_SV_ between the SNCs reflect the shape effects. One possible reason for the relatively smaller *K*_SV_ of the QRs compared to their surface area may be the large curvature along the short axis, which may inhibit binding with HSA. It has been reported that the interaction between proteins and spherical NPs becomes weaker at a smaller NP radius, *i.e.*, a larger surface curvature.^7–10^ In this respect, the smallest *K*_SV_ of the SQDs is likely to be caused by their largest surface curvature.

The inverse of *K*_SV_ for each type of SNC corresponds to a dissociation constant (*K*_D_) in the order of 10 nM. However, this is about three orders of magnitude smaller than the *K*_D_ obtained from FCS experiments in the previous section. Such discrepancies are not uncommon in the binding constants from different techniques. Techniques based on the Trp fluorescence of a protein often provide binding constants in the order of 10^6^ to 10^8^,^7,18,32,59^ whereas those based on the NP radius (FCS or DLS) often fall in the range of 10^3^ to 10^5^.^29,34,51,54^ One possible reason for this discrepancy is that FCS detects the binding of HSA to SNCs from initial to saturation phases, whereas fluorescence quenching may only detect the initial binding of HSA due to the much lower HSA concentration (2.8 μM) compared to that used in FCS.

To elucidate the effect of particle shape on the interaction with HSA, we further investigated this interaction in terms of thermodynamic parameters, namely the binding enthalpy (Δ*H*) and entropy (Δ*S*). For this purpose, similar fluorescence quenching experiments were conducted at various temperatures ranging from 283 to 323 K. At each temperature, the fluorescence spectra were quenched by the SNCs, as shown in Fig. S6, ESI.† The Stern–Volmer plots for the SQDs (Fig. 4(c)) show an increase in the slope (*i.e.*, *K*_SV_) with temperature. Similar trends were also observed for the LQDs and QRs (Fig. S7, ESI†). From the temperature dependence of *K*_SV_, we constructed a van't Hoff plot for each type of SNC, as shown in Fig. 4(d). The van't Hoff equation is as follows:4
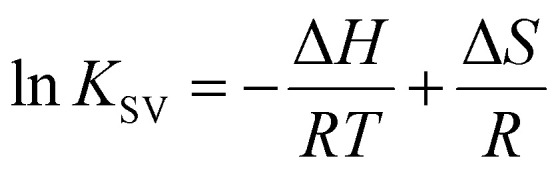
where Δ*H* and Δ*S* are the enthalpy and entropy of the binding of HSA to SNCs, respectively, *R* is the gas constant, and *T* is the absolute temperature. By fitting the plots using eqn (4), Δ*H* and *T*Δ*S* in the Gibbs free energy (Δ*G* = Δ*H* − *T*Δ*S*) were determined at *T* = 298 K and are shown in Table 3.

For each type of SNC, both the Δ*H* and *T*Δ*S* were positive and *T*Δ*S* was much larger than Δ*H*. Therefore, the binding of HSA to SNCs is entropy-driven for the three SNC shapes. Interestingly, among the three types of SNCs, an enthalpy–entropy compensation is visible in the plot of *T*Δ*S vs.* Δ*H* (Fig. 4(e)), satisfying *T*Δ*S* = 0.504 × Δ*H* + 47.5. Such a linear relationship between *T*Δ*S* and Δ*H* has been reported among various protein–NP systems,^31,32^ and enthalpy–entropy compensation is also widely found in protein interactions.^60^ Therefore, the surface of the SNCs used here may have properties similar to those of protein molecules. This is reasonable, considering that the surface of the SNCs in this study was capped by GSH peptides.

In the context of protein–NP interactions, a positive Δ*H* has been interpreted as destruction of the solvating water layer (*i.e.*, desolvation) at the interface, which simultaneously causes a positive entropy change due to the release of water molecules.^31,32^ This process is generally regarded as the hydrophobic interaction, and the latter entropic effects promote binding.^30^ From this viewpoint, the different Δ*H* values among the SNCs in Fig. 4(e) suggest different extents of desolvation upon interaction with HSA. Considering that the surfaces of the three types of SNCs are all covered with GSH and chemically identical (see Fig. 2(c)), their surface areas interacting with HSA are likely to vary, which may be due to the distinct conformations or orientations of the bound HSA, depending on the shape of the SNCs. Consequently, we analyzed the structure of bound HSA using FTIR.

### 3.5. Structure of HSA bound on SNCs

IR spectroscopy can provide information on the secondary structure of proteins through their amide I bands (1600–1800 cm^−1^).^61^ Prior to FTIR measurements, HSA and SNCs were mixed in a molar ratio of 10 : 1. Previous studies have shown that at most 10–20 albumin molecules can be bound to a single NP with a size of 6–7 nm,^51,54^ suggesting that a single SQD (and the larger QR and LQD) would readily accommodate 10 HSA molecules. Furthermore, upon drying for FTIR measurements, the concentration of the HSA–SNC solution became exceptionally high, guaranteeing that all HSA molecules were securely bound to the SNCs.

**Fig. 5:**
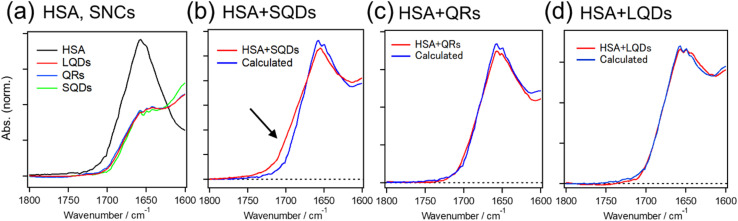
(a) IR spectra of HSA and each type of SNC. The spectra of SNCs are normalized by the amide I band of their surface GSH (∼1643 cm^−1^). (b)–(d) IR spectra of HSA–SNC mixtures (red line) and the fitting results (blue line). The black arrow in (b) indicates the region with the largest fitting error.

Fig. 5 shows the IR spectra of HSA and SNCs, either measured separately (Fig. 5(a)) or in a mixture (Fig. 5(b)–(d)).

The black line in Fig. 5(a) shows the amide I band of HSA at around 1650 cm^−1^, reflecting the dominant α-helix structure of HSA. The overlapping red, blue, and green spectra are due to the surface ligands (GSH) on these three types of SNCs. The red lines in Fig. 5(b)–(d) are IR spectra of the HSA–SQD, HSA–QR, and HSA–LQD complexes, respectively. If HSA does not undergo structural changes upon binding to SNCs, then the IR spectra of the mixed system should match the sum contribution from each component (*i.e.*, IR spectra in Fig. 5(a)). Therefore, we fitted the IR spectra of HSA–SNC complexes using the linear combination of HSA and each type of SNC, *i.e.*, *I*_HSA+SNCs_(*λ*) = *aI*_HSA_(*λ*) + *bI*_SNCs_(*λ*), where *I*_X_(*λ*) is the IR spectrum of component X and *a* and *b* are the fitting parameters. The fitting results are indicated by blue lines in Fig. 5(b)–(d), and they agree well with the experimental spectra of HSA–QR and HSA–LQD (Fig. 5(c) and (d), respectively) but clearly failed for that of HSA–SQD (Fig. 5(b)). These observations indicate that secondary structural changes in HSA only occurred when it was bound to the SQDs. The deviations for HSA–SQD were the largest near 1700 cm^−1^ (black arrow in Fig. 5(b)). This matches the position of the amide I band of the β-sheet or β-turn structure.^61^ In addition, the experimental spectrum deviates positively from the fit in this region. Therefore, we expect that HSA forms β-sheet or β-turn structures concomitant with the partial loss of α-helices upon binding to SQDs. The formation of a β-turn structure during the interaction between albumins and NPs has also been reported using FTIR or circular dichroism (CD) techniques.^19,32,62^

**Fig. 6:**
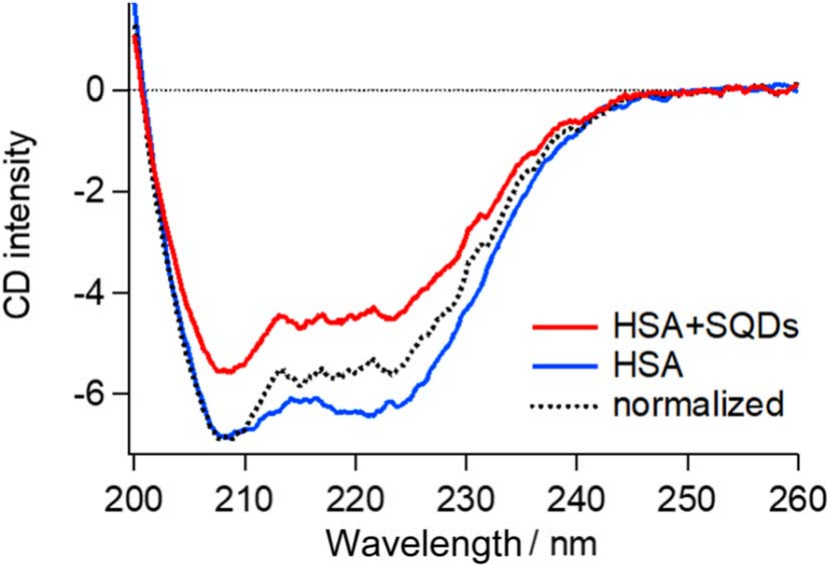
CD spectrum of the HSA–SQD complex (red line) and HSA (blue line) with the same HSA concentration. The black broken line is the spectrum of the HSA–SQD complex normalized with that of HSA at around 208 nm.

To validate the structural changes in HSA upon binding to SQDs, we performed CD measurements on the HSA–SQD complex. Fig. 6 compares the CD spectrum of HSA–SQD with the separately measured CD spectrum of HSA. It's important to note that SQDs themselves exhibited negligible CD signals (data not shown); hence, their contribution can be disregarded.

The concentrations of SQDs and HSA in the mixed solution were 2 μM and 40 μM, respectively. The separately measured HSA solution had the same concentration of 40 μM. The CD spectrum of bound HSA (HSA + SQDs) clearly differed from that of unbound HSA in both intensity and the spectral profile. The normalized spectrum of the HSA–SQD complex in the figure further highlights the spectral changes of HSA upon binding to SQDs.

Secondary structural analysis of the CD spectra revealed a reduction in α-helix content from 65.6% to 46.8% and the emergence of 11% β-sheet content. Additionally, other random and turn structures increased from 34.4% to 42.2%. These changes in the secondary structure are consistent with those observed in IR measurements, confirming the structural changes in HSA upon binding to SQDs.

### 3.6. Shape effects from SNCs on the orientation of bound HSA

The previous section indicated that the varying Δ*H* values for HSA–SNC interactions can be viewed as a representation of the interacting surface area where solvating water molecules are released. Therefore, the contact surface area between HSA and SNC (*S*_SNC–HSA_) should follow the order *S*_SQD–HSA_ > *S*_LQD–HSA_ > *S*_QR–HSA_. However, this seemingly contradicts the fact that the SQDs have the most curved surface, which is unlikely to fit the surface of HSA. FTIR and CD measurements confirmed the formation of β-sheet or β-turn structures in HSA bound to the SQDs. Therefore, it is possible to explain that this structural change affects the shape of HSA molecules and enables them to fit on the curved surface of the SQDs, maximizing *S*_SQD–HSA_. A recent review also pointed out such conformational spreading of proteins adsorbed on a surface through hydrophobic interactions.^6,30^ Additionally, the structural deformability of proteins on the nanoparticle surface has been recently reported.^63,64^ The order *S*_LQD–HSA_ > *S*_QR–HSA_ is presumably because the QRs have a highly curved surface along the short axis that is difficult for HSA to fit, whereas the LQDs have a relatively less curved surface in all directions.

**Fig. 7:**
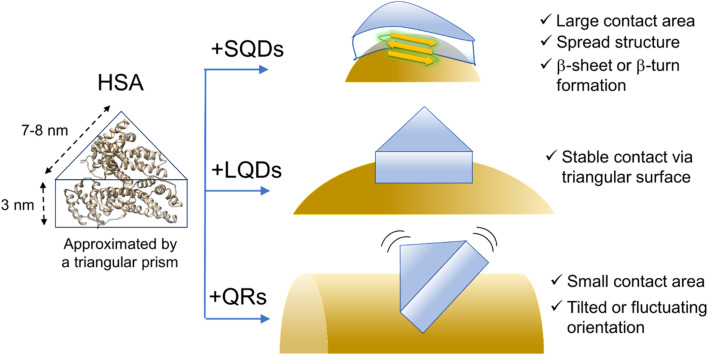
Schematic illustration of the hypothesized structure or conformation of HSA on the SNCs. The surface ligands (GSH) are omitted for clarity. The ribbon structure of HSA was drawn from PDB (1AO6).

Finally, based on the thickness of the protein corona layer estimated from the FCS experiments (Δ*d* in Table 2), the orientation or structure of HSA molecules on the SNCs can be discussed. Δ*d* values of SQDs (2.9 nm) or LQDs (3.2 nm) were close to the expected height of HSA (3 nm) from its structure. Therefore, the HSA molecule is thought to lie flat on the surface of SQDs or LQDs and form stable contact *via* its triangular face. In the case of SQDs, HSA undergoes structural modifications as previously demonstrated, which may be attributed to the snug fit of the triangular face of HSA onto the curved surface of SQDs, resulting in its largest contact area (*S*_SQD–HSA_). In contrast, in the case of QRs, Δ*d* showed clearly a larger value (4.6 nm) than the height of HSA. This larger Δ*d* value can be explained by assuming that HSA molecules “stand” on the surface of QRs *via* the edge of its prism-like shape, which is consistent with the smaller contact area (*S*_QR–HSA_) previously concluded for the HSA–QRs interactions. On the basis of these speculations, the predicted orientation of HSA on the three types of SNCs is schematically shown in Fig. 7.

Fig. 7 shows that HSA recognizes the local surface curvature of each type of SNC and adopts an appropriate conformation or structure. Indeed, Kahn *et al.* performed docking simulation between HSA and differently sized gold NPs^10^ and found that the bound HSA alters its conformation in response to the NP size (*i.e.*, surface curvature). Specifically, those authors found that for smaller NPs (4–10 nm) the triangular prism-shaped HSA molecule “stands” on the NP surface *via* its lateral face. For larger NPs (11–20 nm), the triangular face of HSA is used for binding to the NPs instead. These docking geometries are consistent with Fig. 7 here, since the short axis length of the QRs is within 4–10 nm whereas the size of the LQDs is within 11–20 nm. Kahn *et al.* also showed that the stabilization energy for binding increased by approximately 15 kcal mol^−1^ as the radius of gold NPs increased from 7 nm (comparable to the SQDs) to 15 nm (comparable to the LQDs). This may explain that the SQDs induce a secondary structural change in HSA to gain stabilization energy.

## 4. Conclusion

To elucidate the effects of NP shape on protein corona formation, three types of SNCs of different shapes (SQDs, LQDs, and QRs) were synthesized, and their interactions with HSA were examined using various spectroscopic methods (FCS, fluorescence quenching, and FTIR) combined with thermodynamic analysis. The shape of the SNCs affects their interaction with HSA in terms of binding strength or thermodynamic parameters. The results consistently showed that the conformation or orientation of bound HSA molecules depended on the morphology of the SNCs. This dependency was explained by the local surface curvature “sensed” by HSA. Our study demonstrated how the surface curvature of NPs affects the binding mode of a protein, providing a mechanistic view of the effects of NP shape on protein corona formation.

Beyond the shapes of nanoparticles, the shape, size, and deformability of the protein itself can also significantly influence protein corona formation.^64–66^ Indeed, we are currently investigating the shape effects of adsorbed proteins, which will be published as a separate study in the future. A comprehensive understanding of the individual and combined roles of nanoparticle and protein shape in protein corona formation will ultimately guide the development of more effective nanomedicines.

## Abbreviations

FCS: fluorescence correlation spectroscopy
FTIR: Fourier transform infrared spectroscopy
GSH: glutathione
HSA: human serum albumin
LQDs: large quantum dots
QRs: quantum rods
SNCs: semiconductor nanocrystals
SQDs: small quantum dots

## Data availability

The authors confirm that the data supporting the findings of this study are available within the article and its ESI.† All datasets used in the current study are available from the corresponding author upon reasonable request.

## Conflicts of interest

There are no conflicts of interest to declare.

## Supplementary Material

NA-007-D4NA00696H-s001
