# Supplementary material for: Protein corona formation on different-shaped CdSe/CdS semiconductor nanocrystals
Source: Nanoscale Adv. 2024 Nov 25;7(2):560–71. doi: 10.1039/d4na00696h (PMC11621831; doi:10.1039/d4na00696h)
Supplement: NA-007-D4NA00696H-s001 [file NA-007-D4NA00696H-s001.pdf]

Supplementary Information (SI) for Nanoscale Advances.
This journal is © The Royal Society of Chemistry 2024

Electric Supplementary Information

Protein corona formation on different-shaped CdSe/CdS semiconductor nanocrystals

Kunisato Kuroi*,[a] Yuta Kanazawa,[a] Akane Shinaridome[a], Yuna Yasuda,[a] Minkyo Jung,[b] Chan-Gi Pack[c,d] and Fumihiko Fujii*[a]

[a]*Faculty of Pharmaceutical Sciences, Kobe Gakuin University, Kobe 650-8586, Japan*

[b]*Neural Circuit Research Group, Korea Brain Research Institute, Daegu 41062, Korea*

[c]*Convergence Medicine Research Center (CREDIT), Asan Institute for Life Sciences, Asan Medical Center, Seoul 05505, Korea*

[d]*Department of Convergence Medicine, University of Ulsan College of Medicine, Seoul 05505, Korea*

S-1. Statistical analysis of SNCs particle size from TEM images

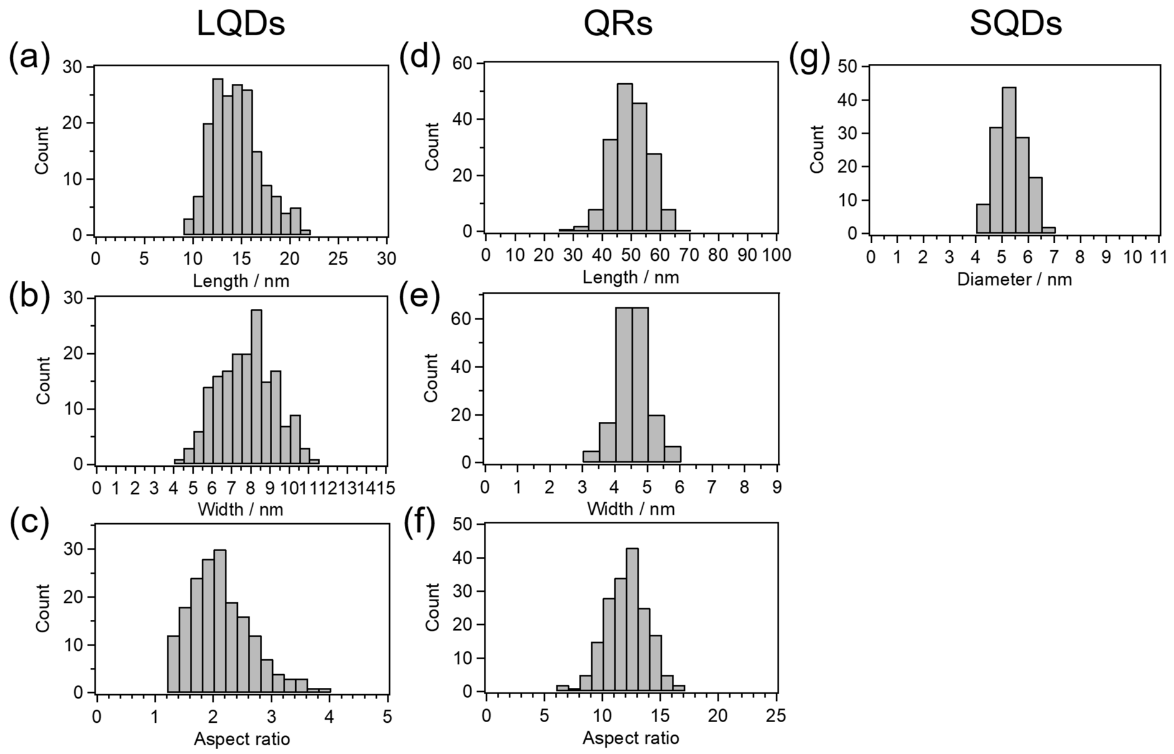

Fig. S1 Size distributions of the LQDs in (a) length (L), (b) width (W), and (c) aspect ratio (p). Size distributions of the QRs in (d) length (L), (e) width (W), and (f) aspect ratio (p). (g) Size distribution of the SQDs in diameter (d). For each type of SNCs, about 150 particles from TEM images were used in statistical analysis.

Table S1 Averaged dimensions of the SNCs in Fig. S1.

	QRs	LQDs	SQDs
W (or d) / nm	4.05±0.52	7.30±1.51	4.85±0.60
L / nm	44.32±6.60	13.51±2.60	-
$p = L / W$	11.08±1.93	1.93±0.56	-

S-2. Modified Stokes-Einstein equation for football- and rod-shaped particles

The diffusion coefficient (D) of a spherical solute of diameter of d is given by the normal Stokes-Einstein (SE) equation:

$$D = \frac{k_B T}{3\pi\eta d} \qquad \text{(S1)}$$

where k_B is Boltzmann's constant, T is the absolute temperature, and η is the solvent viscosity. In DLS measurements, the hydrodynamic diameter (d) of a particle is calculated from its diffusion coefficient and the SE equation above, assuming that all particles are spherical.

When the solute is a football- or rod-shaped particle with a long axis length (L) and a short axis length (W), the SE equation is modified as follows:[1,2]

$$D = \frac{k_B T}{3\pi\eta d_{\mathrm{H}}(L, W)} \qquad \text{(S2)}$$

with

$$d_{\mathrm{H}}(L, W) = \frac{\sqrt{L^2 - W^2}}{\ln\left(\frac{L + \sqrt{L^2 - W^2}}{W}\right)} = W \frac{\sqrt{p^2 - 1^2}}{\ln\left(p + \sqrt{p^2 - 1^2}\right)} \qquad \text{(S3)}$$

where d_{H} is the hydrodynamic diameter measured using DLS, p is the aspect ratio ($p = L/W$). By substituting the L and W values in Table 1 into eqn (S3), the d_{H} values for the LQDs and QRs were calculated to be 11.48 and 17.10 nm, respectively.

S-3. Analysis of the absorption spectrum of QRs to deduce the rod width

The red line in Fig. S2 shows the tangent at the inflection point of the absorption peak of CdS shell of the QRs. Its x-intercept is 469.6 nm. This x-intercept (λ_t) has been reported to correlate with the width (W) of bare CdSe/CdS nanorods.[3] According to the same study, this correlation is experimentally described as follows:

$$W(E) = \frac{2.62 \times 10^{-36}}{\sqrt{(E - 3.69 \times 10^{-19}) \cdot 5.24 \times 10^{-36} + 4.60 \times 10^{-57}} + 6.79 \times 10^{-29}} \quad \text{(S4)}$$

where E is the energy (J) corresponding to the x-intercept in wavelength, $E = hc/\lambda_t$. By converting λ_t = 469.9 nm to $E = 4.23 \times 10^{-19}$ J and substituting the latter into eqn (S4), the rod width of bare QRs was calculated to be W = 4.33 nm.

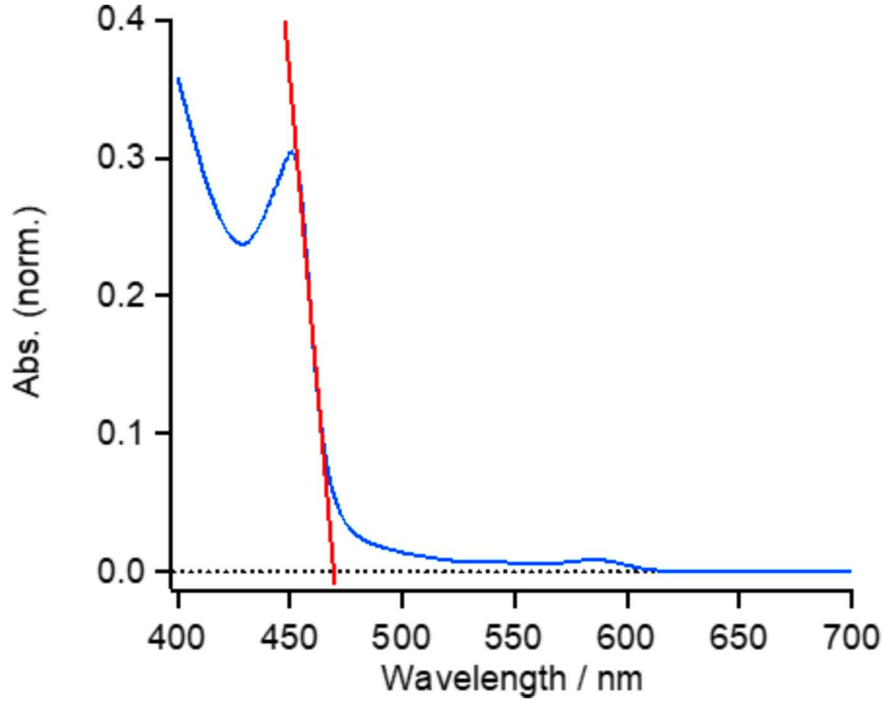

Fig. S2 UV-Vis absorption spectrum of the QRs (blue line) and the tangent line (red line). The tangent line is drawn around the inflection point of the CdS shell peak on the longer wavelength side.

S-4. Quantum yield (QY) of the SNCs

The fluorescence quantum yield (QY) of the solubilized SNCs was determined by measuring the UV-Vis and fluorescence spectra of SNCs at several concentrations. The integrated fluorescence intensity (I) and absorbance (A) at an excitation wavelength of 480 nm were plotted to obtain the QY using the following analysis. If m is the proportional constant between I and A (i.e., $I = mA$), then it represents the relative value of QY. Rhodamine 6G (R6G) in ethanol was used as the reference dye and measured under the same conditions. Because m of R6G corresponds to the literature QY value ($QY_{\mathrm{R6G}} = 0.94$), the QY of the SNCs (QY_{SNCs}) can be calculated as follows:[4]

$$QY_{\mathrm{SNCs}} = QY_{\mathrm{R6G}} \frac{m_{\mathrm{SNCs}}}{m_{\mathrm{R6G}}} \left(\frac{n_{\mathrm{water}}}{n_{\mathrm{ethanol}}} \right)^2 \qquad \text{(S5)}$$

where n represents the refractive index of solvents.

Figure S3 shows the I–A plots for each type of SNCs. From these plots, the m values were determined using eqn (S5) to be 2.86×10^9 for the SQDs, 2.12×10^9 for the QRs, and 3.47×10^8 for the LQDs. Similarly, m of R6G was determined to be 5.43×10^9 (plot not shown). Using these values, the QY of each type of SNCs was determined to be 0.47 (SQDs), 0.35 (QRs), and 0.06 (LQDs).

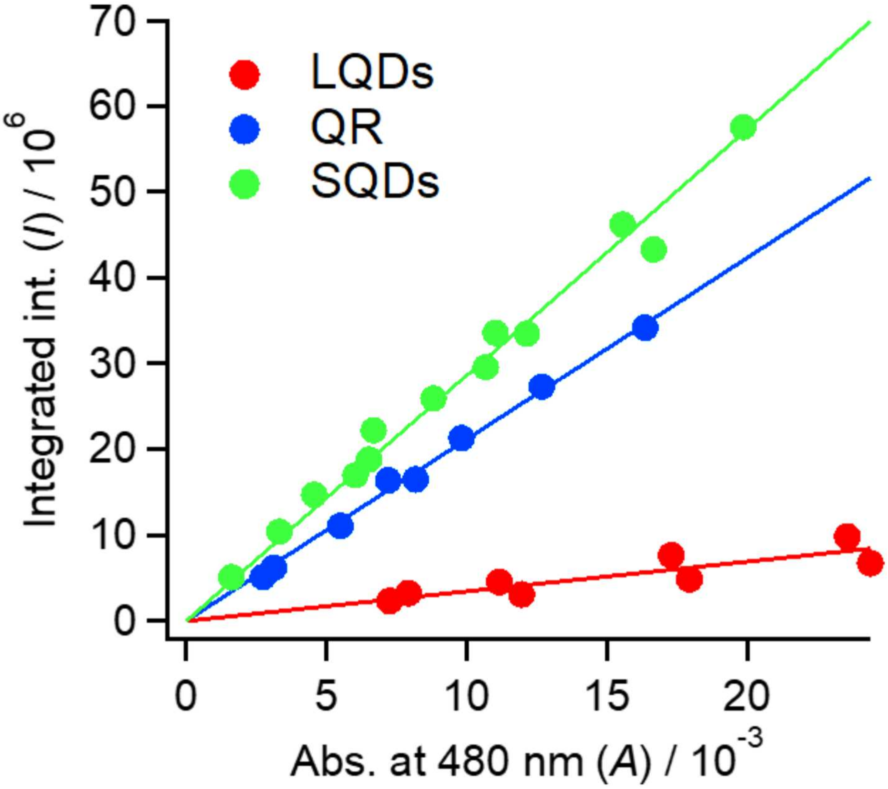

Fig. S3 Plots of the integrated fluorescent intensity (I) versus the absorbance at 480 nm excitation wavelength (A) for each type of SNCs. Solid lines: linear fits to $I = mA$ to obtain m using eqn (S5).

S-5. Typical fitting results of autocorrelation curves in FCS measurements of SNCs

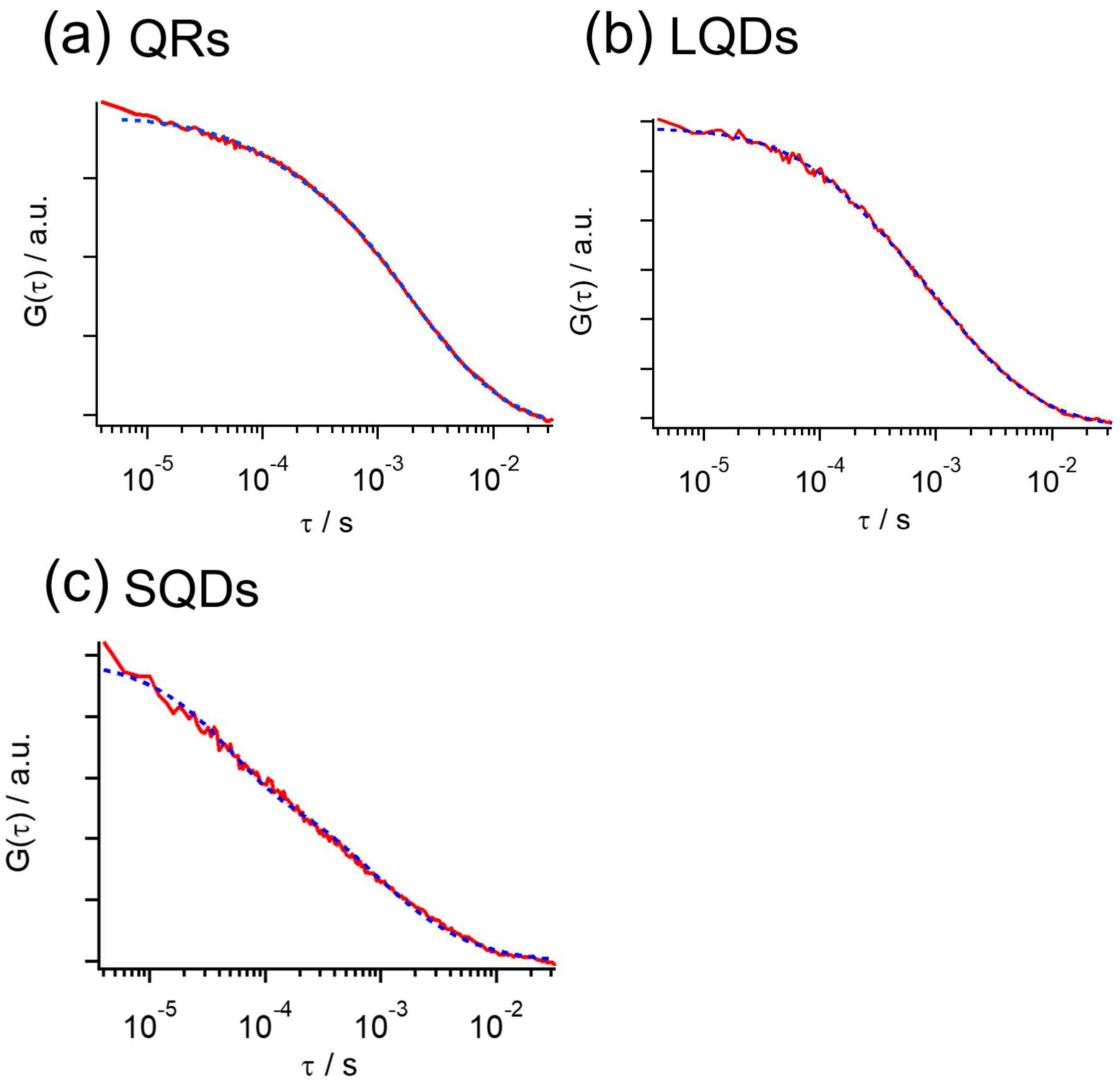

Fig. S4 Fitting results of autocorrelation curves ($G(\tau)$) in FCS measurements of the SNCs: (a) QRs, (b) LQDs, and (c) SQDs. HSA concentration: 100 μM, red solid lines: experimental data, blue dashed lines: fitting curves using eqn (1).

S-6. Derivation of eqn (2)

Suppose N molecules of HSA bind to an SNC when the HSA concentration is x and the maximum number of bound HSA molecules per particle is N_{max}. The binding process is further assumed to be described by the Hill equation with the apparent dissociation constant K_D and the Hill coefficient n:

$$N = N_{max}\frac{x^n}{x^n + K_D^n} \qquad (S6)$$

When N molecules of HSA bind to the SNC, the particle volume (V_0) increases by a factor of ($1+cN$), where c is an appropriate constant. The new particle volume $V(N)$ can then be written as:

$$V(N) = (1 + cN)V_0 \qquad (S7)$$

For the spherical SQDs, the hydrodynamic diameter $d_H(N)$ is proportional to $\sqrt[3]{V(N)}$. This is also true for the nonspherical LQDs and QRs, assuming that their aspect ratios ($p = L/W$ in Section S-2) are unchanged. This approximation is valid to some extent because previous studies showed that HSA adsorbs onto SNCs as a monolayer.[5,6] When p is regarded as a constant, the d_H of the LQDs or QRs (eqn (S3)) is proportional to the particle dimension (L or W), which is proportional to $\sqrt[3]{V(N)}$. From the proportionality between $d_H(N)$ and $\sqrt[3]{V(N)}$, we obtain eqn (S8) from eqn (S7) as:

$$d_H(N) = d_H(0)\sqrt[3]{(1 + cN)} \qquad (S8)$$

This relationship was previously demonstrated by Rocker et al.[6] and assumed to be applicable to the nonspherical QRs and LQDs.

By substituting eqn (S6) into eqn (S8), d_H can be written as a function of the HSA concentration (x):

$$d_H(x) = d_H(0)\left(1 + C\frac{x^n}{x^n + K_{\mathrm{D}}^n}\right)^{\frac{1}{3}} \qquad \text{(S9)}$$

where $C = cN_{\mathrm{max}}$. Because d_{H} is inversely proportional to D, by inverting both sides of eqn (S9), we obtain D as a function of x:

$$D(x) = D_0\left(1 + C\frac{x^n}{x^n + K_{\mathrm{D}}^n}\right)^{-\frac{1}{3}} \qquad \text{(S10)}$$

When $x >> K_{\mathrm{D}}$, the binding of HSA is saturated and D is assumed to be close to the plateau value (D_{min}). Because K_{D} is negligible compared to x, we obtain

$$D_{min} = D_0(1 + C)^{-\frac{1}{3}}$$

By eliminating the constant C in eqn (S10) using this relationship, we obtain eqn (2) in the main text:

$$D(x) = D_0\left\{1 + \left(\left(\frac{D_0}{D_{min}}\right)^3 - 1\right)\left(\frac{x^n}{x^n + K_d^n}\right)\right\}^{-\frac{1}{3}}$$

S-7. Fluorescence spectra of HSA quenched by QRs or LQDs

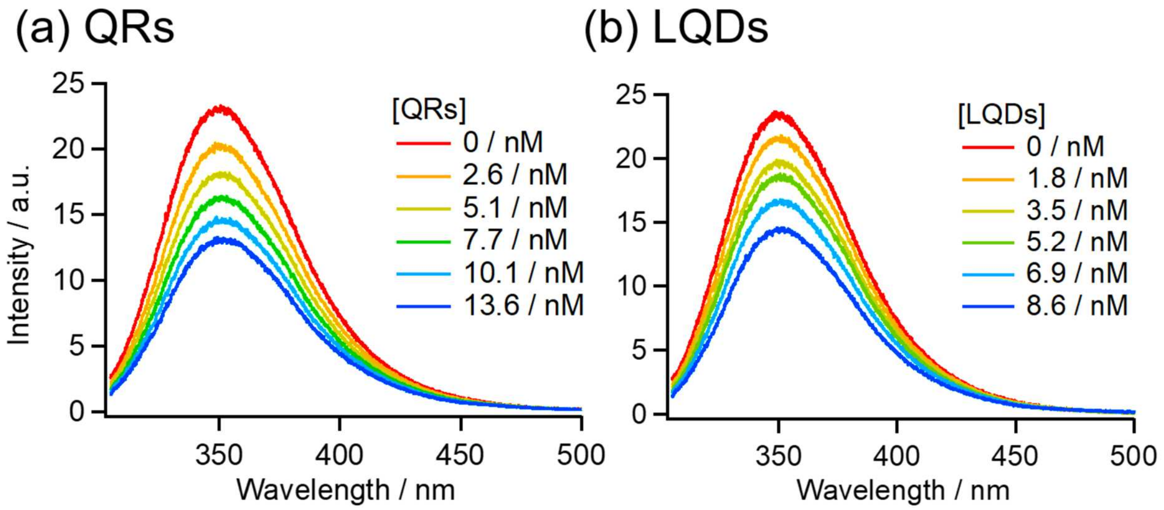

Fig. S5 Quenching of HSA fluorescence by (a) QRs and (b) LQDs. The fluorescence spectra were measured at room temperature (298 K) and averaged over at least 3 independent experiments.

S-8. Fluorescence spectra of HSA quenched by SNCs at various temperatures

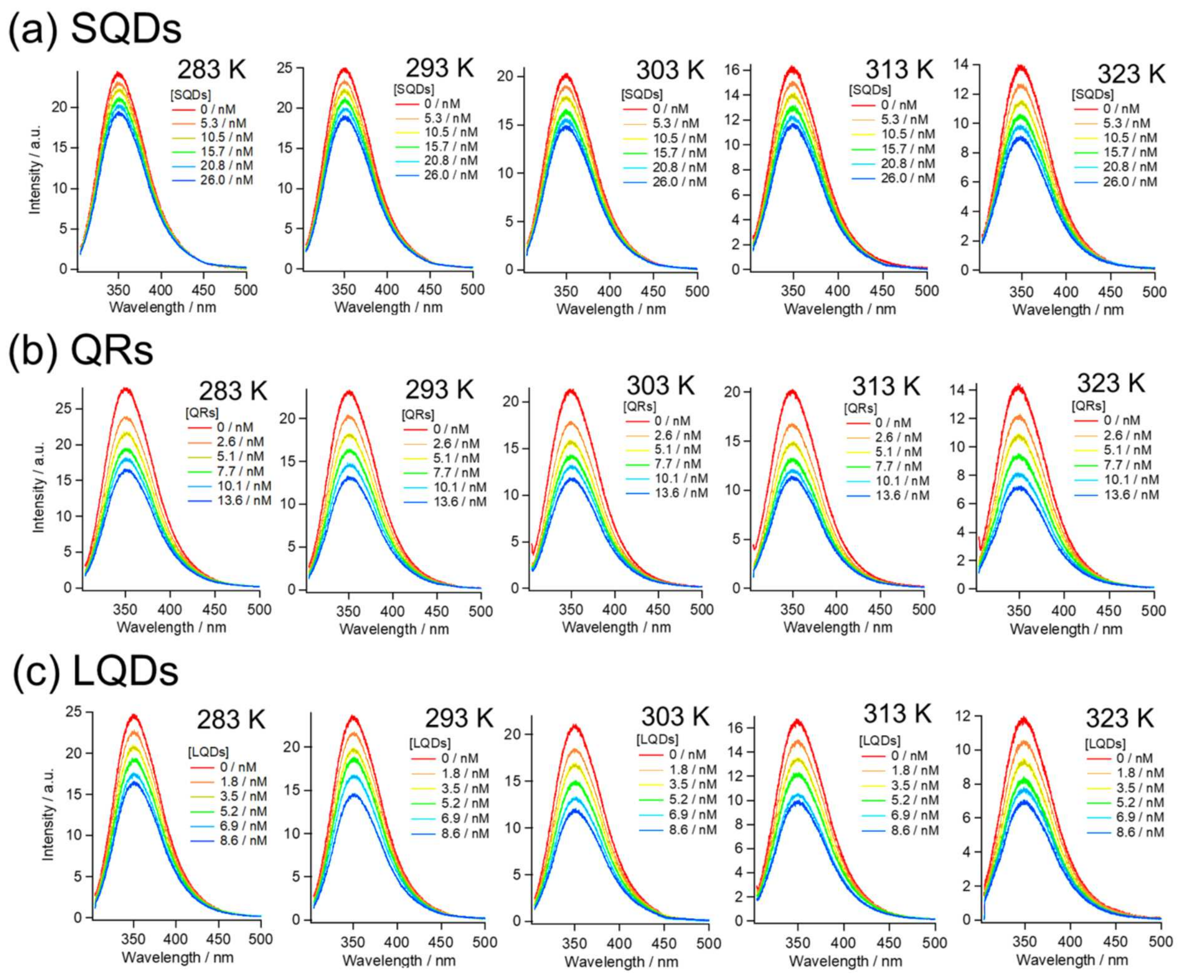

Fig. S6 Quenching of HSA fluorescence by SNCs under various temperatures from 283 to 323 K for (a) SQDs, (b) QRs, and (c) LQDs. The spectra were averaged over at least 3 independent experiments.

S-9. Stern-Volmer plots for QRs and LQDs at various temperatures

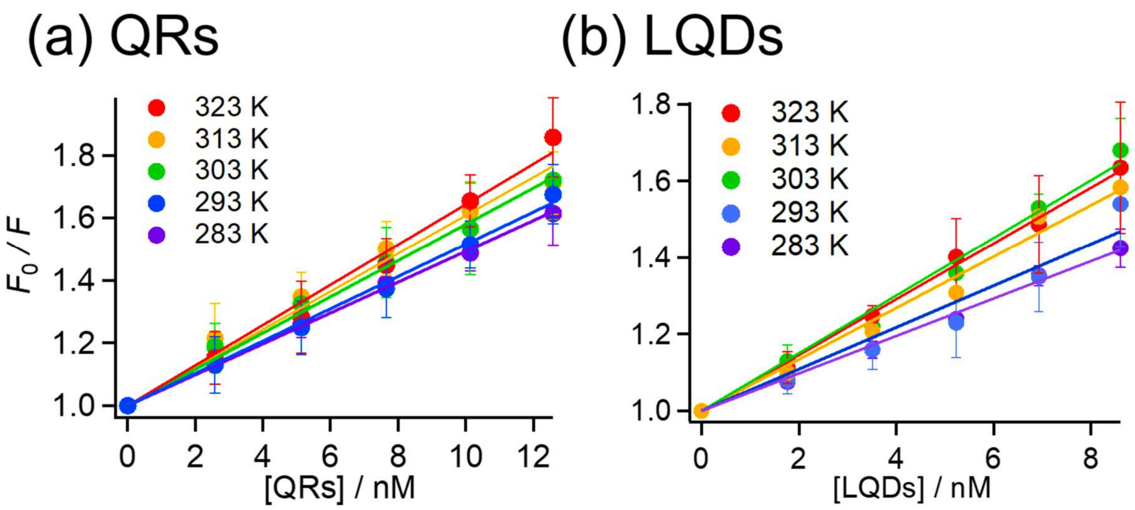

Fig. S7 Stern-Volmer plots for (a) QRs and (b) LQDs at temperatures from 283 to 323 K.

REFERENCES

(1) E. L. Cussler, *Diffusion (mass transfer in fluid systems)*, 3rd ed.; Cambridge, 2009, pp 129.

(2) D. Feller, M. Otten, M. Hildebrandt, M. Krüsmann, G. Bryant, and M. Karg, Translational and rotational diffusion coefficients of gold nanorods functionalized with a high molecular weight, thermoresponsive ligand: a depolarized dynamic light scattering study, *Soft matter*, 2021, **17**, 4019–4026.

(3) P. Abel, J. Bloh, D. Hinrichs, T. Kodanek, and D. Dorfs, Determination of all dimensions of CdSe seeded CdS nanorods solely via their UV/Vis spectra, *Z. Phys. Chem.*, 2017, **231**, 93–106.

(4) J.R. Lakowicz, *Principles of Fluorescence Spectroscopy*, 3rd ed.; Springer, 2006, pp 54–55.

(5) L. Shang and G. U. Nienhaus, *Acc. Chem. Res.*, 2017, **50**, 387–395.

(6) C. Röcker, M. Pötzl, F. Zhang, W. J. Parak and G. U. Nienhaus, A quantitative fluorescence study of protein monolayer formation on colloidal nanoparticles, *Nat. Nanotechnol.*, 2009, **4**, 577–580.
